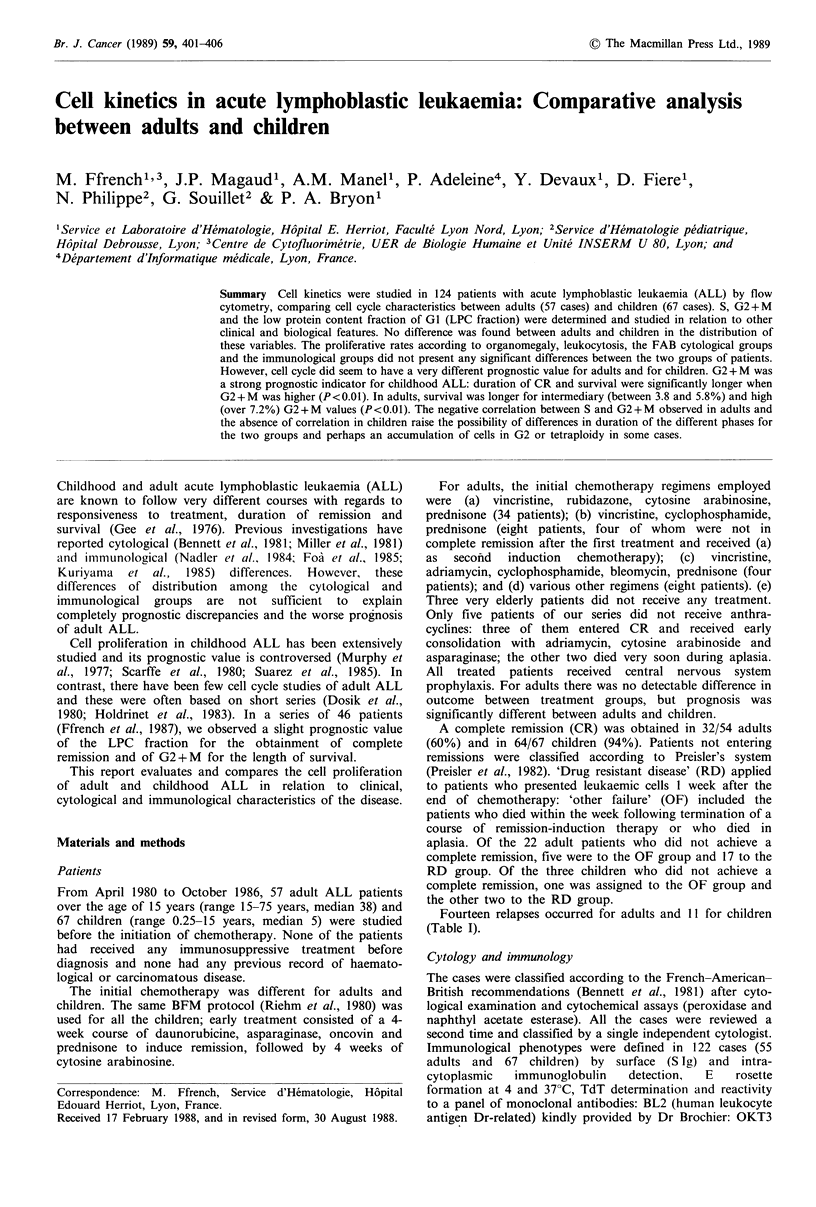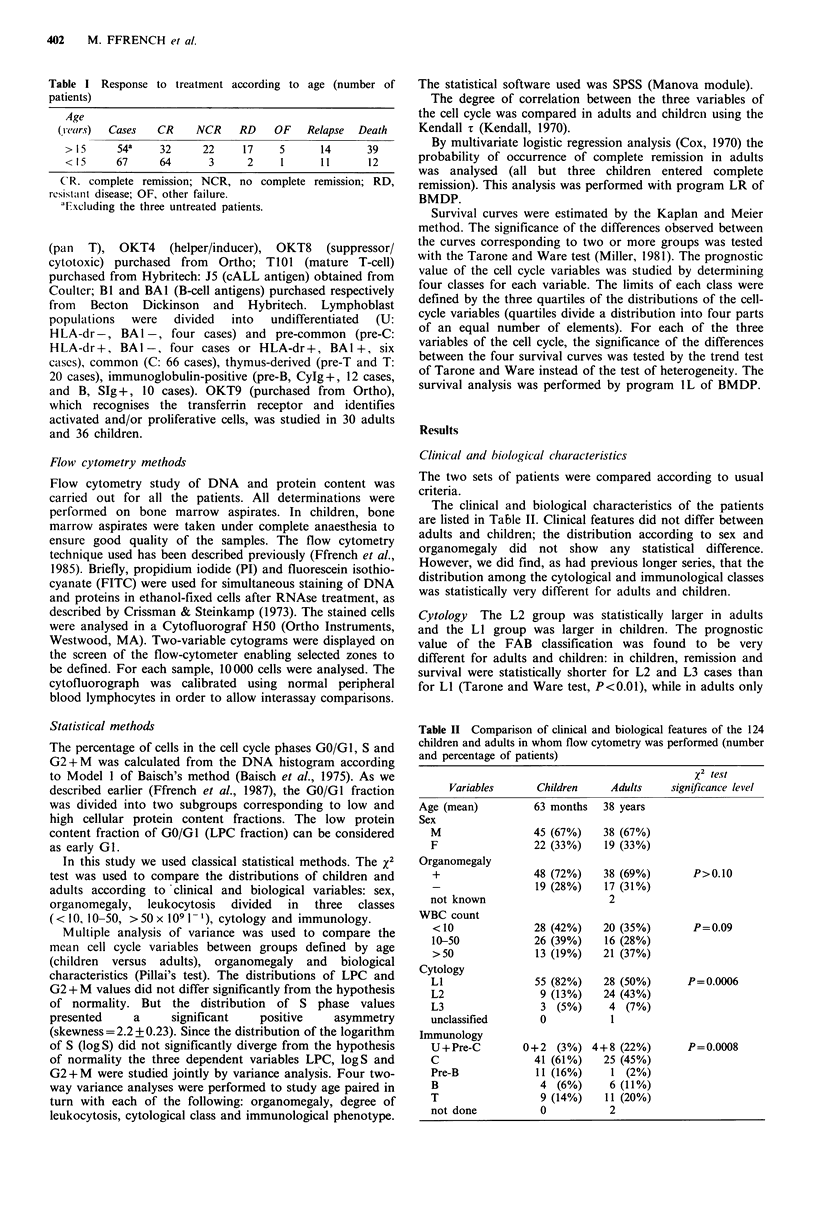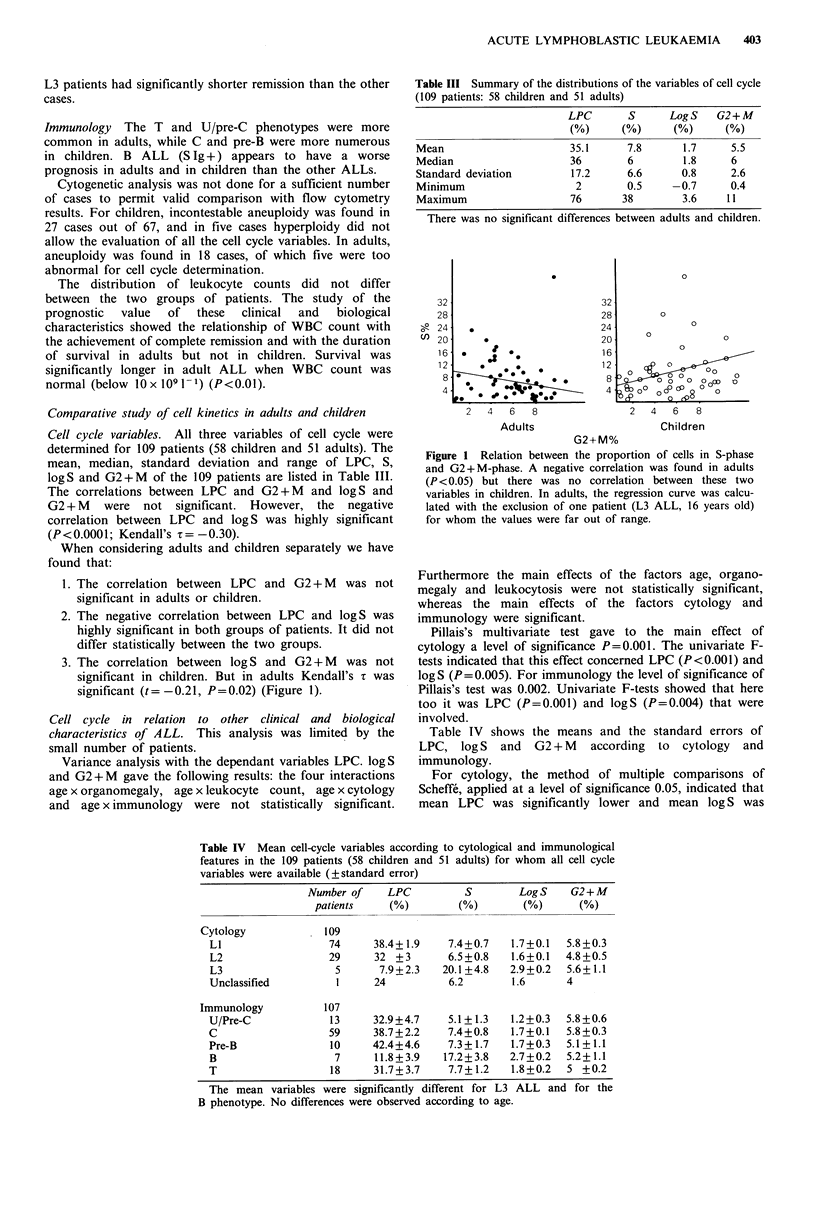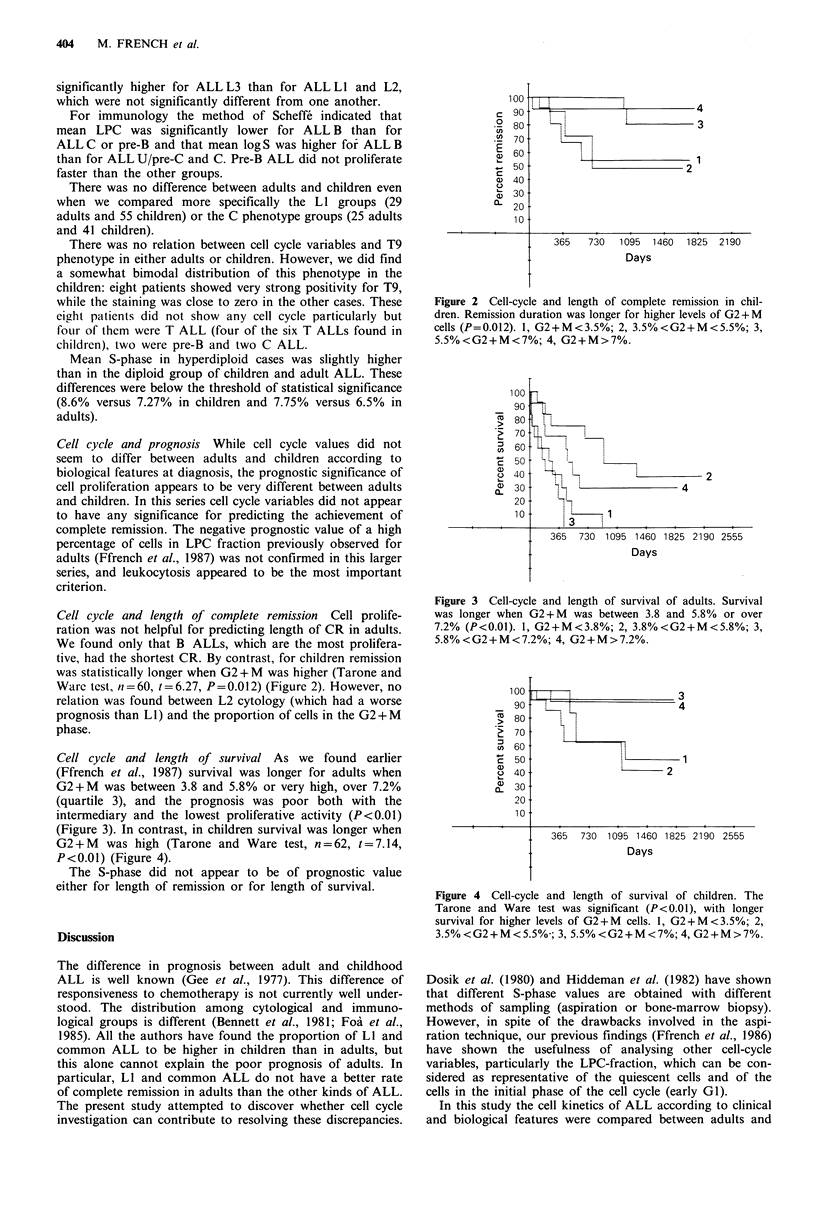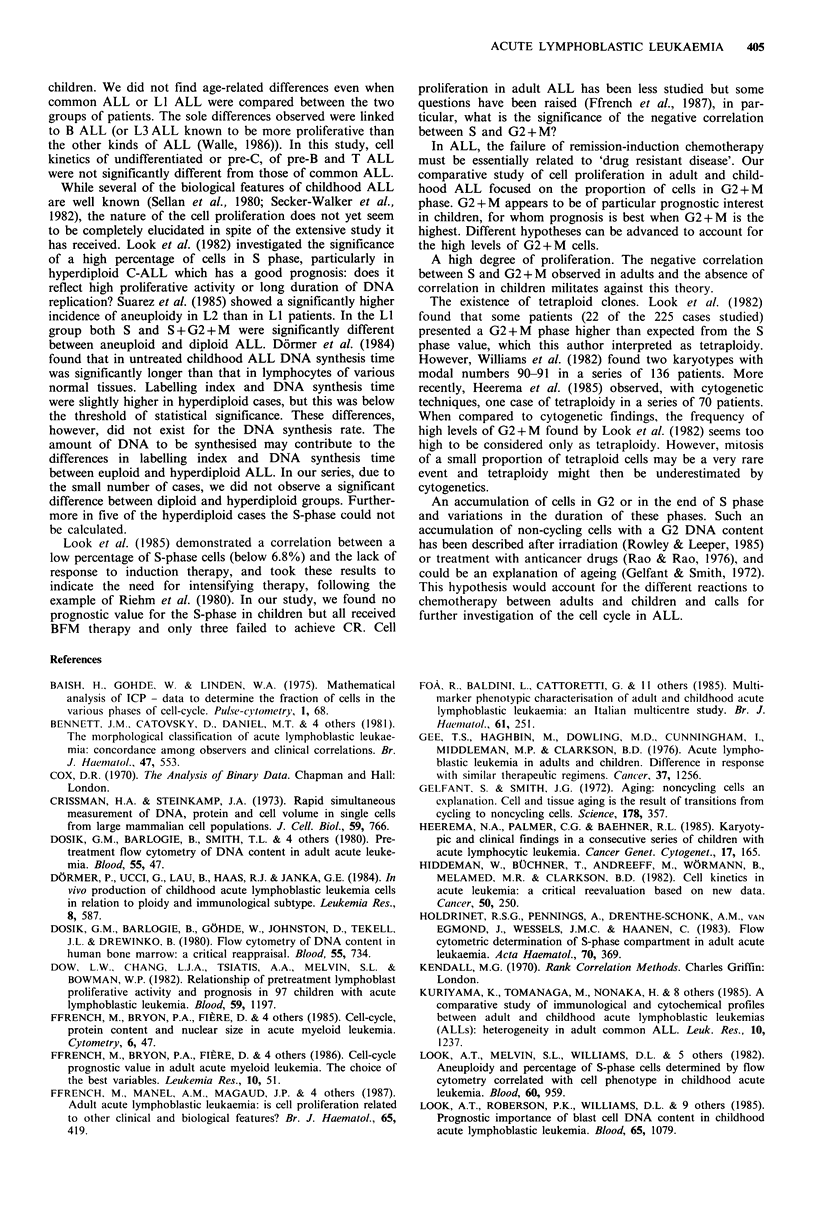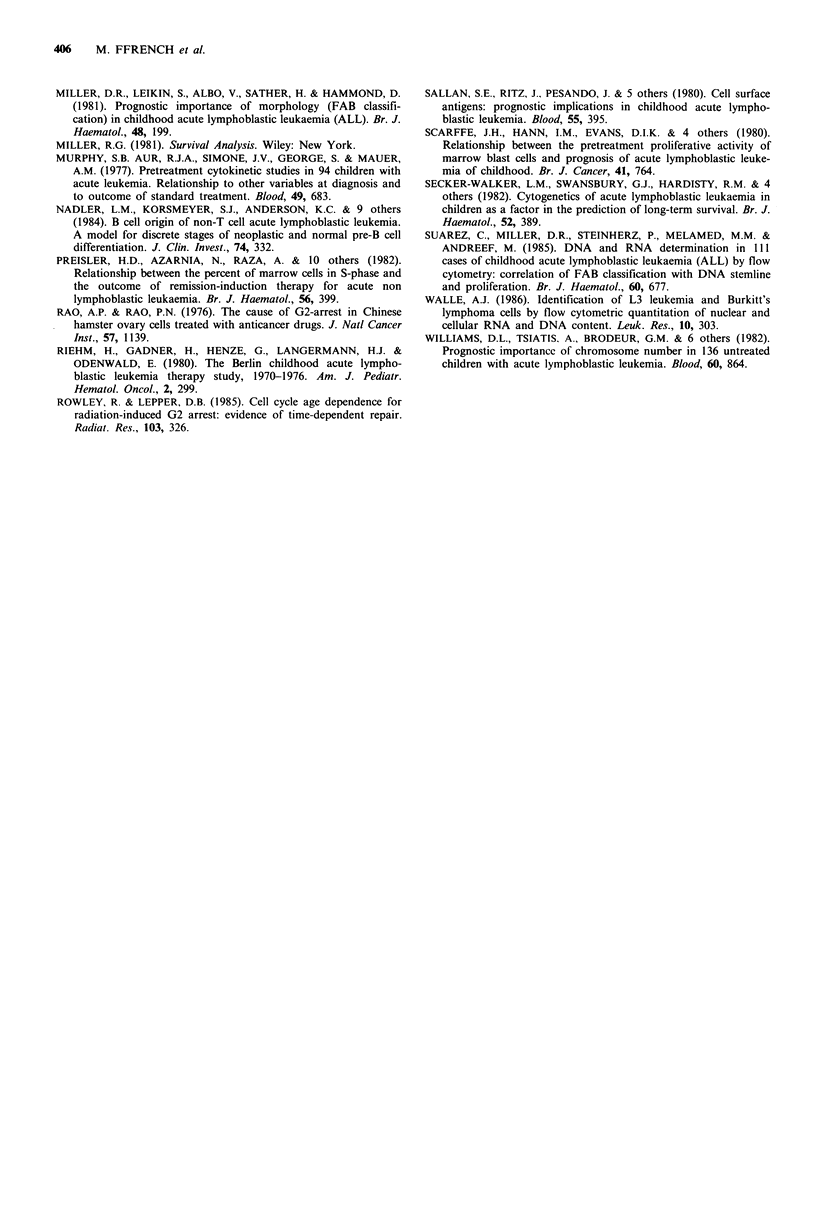# Cell kinetics in acute lymphoblastic leukaemia: comparative analysis between adults and children.

**DOI:** 10.1038/bjc.1989.80

**Published:** 1989-03

**Authors:** M. Ffrench, J. P. Magaud, A. M. Manel, P. Adeleine, Y. Devaux, D. Fiere, N. Philippe, G. Souillet, P. A. Bryon

**Affiliations:** Service et Laboratoire d'HÃ©matologie, HÃ´pital E. Herriot, FacultÃ© Lyon Nord, France.

## Abstract

Cell kinetics were studied in 124 patients with acute lymphoblastic leukaemia (ALL) by flow cytometry, comparing cell cycle characteristics between adults (57 cases) and children (67 cases). S, G2 + M and the low protein content fraction of G1 (LPC fraction) were determined and studied in relation to other clinical and biological features. No difference was found between adults and children in the distribution of these variables. The proliferative rates according to organomegaly, leukocytosis, the FAB cytological groups and the immunological groups did not present any significant differences between the two groups of patients. However, cell cycle did seem to have a very different prognostic value for adults and for children. G2 + M was a strong prognostic indicator for childhood ALL: duration of CR and survival were significantly longer when G2 + M was higher (P less than 0.01). In adults, survival was longer for intermediary (between 3.8 and 5.8%) and high (over 7.2%) G2 + M values (P less than 0.01). The negative correlation between S and G2 + M observed in adults and the absence of correlation in children raise the possibility of differences in duration of the different phases for the two groups and perhaps an accumulation of cells in G2 or tetraploidy in some cases.


© The Macmillan Press Ltd., 1989

### Cell kinetics in acute lymphoblastic leukaemia: Comparative analysis between adults and children

M. Ffrench[1,3], J.P. Magaud[1], A.M. Manel[1], P. Adeleine[4], Y. Devaux[1], D. Fiere[1], N. Philippe[2], G. Souillet[2] & P. A. Bryon[1]

[1]*Service et Laboratoire d'Hématologie, Hôpital E. Herriot, Faculté Lyon Nord, Lyon;* [2]*Service d'Hématologie pédiatrique, Hôpital Debrousse, Lyon;* [3]*Centre de Cytofluorimétrie, UER de Biologie Humaine et Unité INSERM U 80, Lyon; and* [4]*Département d'Informatique médicale, Lyon, France.*

**Summary** Cell kinetics were studied in 124 patients with acute lymphoblastic leukaemia (ALL) by flow cytometry, comparing cell cycle characteristics between adults (57 cases) and children (67 cases). S, G2+M and the low protein content fraction of G1 (LPC fraction) were determined and studied in relation to other clinical and biological features. No difference was found between adults and children in the distribution of these variables. The proliferative rates according to organomegaly, leukocytosis, the FAB cytological groups and the immunological groups did not present any significant differences between the two groups of patients. However, cell cycle did seem to have a very different prognostic value for adults and for children. G2+M was a strong prognostic indicator for childhood ALL: duration of CR and survival were significantly longer when G2+M was higher ($P<0.01$). In adults, survival was longer for intermediary (between 3.8 and 5.8%) and high (over 7.2%) G2+M values ($P<0.01$). The negative correlation between S and G2+M observed in adults and the absence of correlation in children raise the possibility of differences in duration of the different phases for the two groups and perhaps an accumulation of cells in G2 or tetraploidy in some cases.

Childhood and adult acute lymphoblastic leukaemia (ALL) are known to follow very different courses with regards to responsiveness to treatment, duration of remission and survival (Gee *et al.*, 1976). Previous investigations have reported cytological (Bennett *et al.*, 1981; Miller *et al.*, 1981) and immunological (Nadler *et al.*, 1984; Foà *et al.*, 1985; Kuriyama *et al.*, 1985) differences. However, these differences of distribution among the cytological and immunological groups are not sufficient to explain completely prognostic discrepancies and the worse prognosis of adult ALL.

Cell proliferation in childhood ALL has been extensively studied and its prognostic value is controversed (Murphy *et al.*, 1977; Scarffe *et al.*, 1980; Suarez *et al.*, 1985). In contrast, there have been few cell cycle studies of adult ALL and these were often based on short series (Dosik *et al.*, 1980; Holdrinet *et al.*, 1983). In a series of 46 patients (Ffrench *et al.*, 1987), we observed a slight prognostic value of the LPC fraction for the obtainment of complete remission and of G2+M for the length of survival.

This report evaluates and compares the cell proliferation of adult and childhood ALL in relation to clinical, cytological and immunological characteristics of the disease.

### Materials and methods

### *Patients*

From April 1980 to October 1986, 57 adult ALL patients over the age of 15 years (range 15–75 years, median 38) and 67 children (range 0.25–15 years, median 5) were studied before the initiation of chemotherapy. None of the patients had received any immunosuppressive treatment before diagnosis and none had any previous record of haematological or carcinomatous disease.

The initial chemotherapy was different for adults and children. The same BFM protocol (Riehm *et al.*, 1980) was used for all the children; early treatment consisted of a 4-week course of daunorubicine, asparaginase, oncovin and prednisone to induce remission, followed by 4 weeks of cytosine arabinosine.

Correspondence: M. Ffrench, Service d'Hématologie, Hôpital Edouard Herriot, Lyon, France.
Received 17 February 1988, and in revised form, 30 August 1988.

For adults, the initial chemotherapy regimens employed were (a) vincristine, rubidazone, cytosine arabinosine, prednisone (34 patients); (b) vincristine, cyclophosphamide, prednisone (eight patients, four of whom were not in complete remission after the first treatment and received (a) as second induction chemotherapy); (c) vincristine, adriamycin, cyclophosphamide, bleomycin, prednisone (four patients); and (d) various other regimens (eight patients). (e) Three very elderly patients did not receive any treatment. Only five patients of our series did not receive anthracyclines: three of them entered CR and received early consolidation with adriamycin, cytosine arabinoside and asparaginase; the other two died very soon during aplasia. All treated patients received central nervous system prophylaxis. For adults there was no detectable difference in outcome between treatment groups, but prognosis was significantly different between adults and children.

A complete remission (CR) was obtained in 32/54 adults (60%) and in 64/67 children (94%). Patients not entering remissions were classified according to Preisler's system (Preisler *et al.*, 1982). 'Drug resistant disease' (RD) applied to patients who presented leukaemic cells 1 week after the end of chemotherapy: 'other failure' (OF) included the patients who died within the week following termination of a course of remission-induction therapy or who died in aplasia. Of the 22 adult patients who did not achieve a complete remission, five were to the OF group and 17 to the RD group. Of the three children who did not achieve a complete remission, one was assigned to the OF group and the other two to the RD group.

Fourteen relapses occurred for adults and 11 for children (Table I).

### *Cytology and immunology*

The cases were classified according to the French–American–British recommendations (Bennett *et al.*, 1981) after cytological examination and cytochemical assays (peroxidase and naphthyl acetate esterase). All the cases were reviewed a second time and classified by a single independent cytologist. Immunological phenotypes were defined in 122 cases (55 adults and 67 children) by surface (S Ig) and intracytoplasmic immunoglobulin detection, E rosette formation at 4 and 37°C, TdT determination and reactivity to a panel of monoclonal antibodies: BL2 (human leukocyte antigen Dr-related) kindly provided by Dr Brochier: OKT3

**Table I** Response to treatment according to age (number of patients)

| Age (years) | Cases | CR | NCR | RD | OF | Relapse | Death |
|---|---|---|---|---|---|---|---|
| >15 | 54[a] | 32 | 22 | 17 | 5 | 14 | 39 |
| <15 | 67 | 64 | 3 | 2 | 1 | 11 | 12 |

CR, complete remission; NCR, no complete remission; RD, resistant disease; OF, other failure.
[a]Excluding the three untreated patients.

(pan T), OKT4 (helper/inducer), OKT8 (suppressor/cytotoxic) purchased from Ortho; T101 (mature T-cell) purchased from Hybritech: J5 (cALL antigen) obtained from Coulter; B1 and BA1 (B-cell antigens) purchased respectively from Becton Dickinson and Hybritech. Lymphoblast populations were divided into undifferentiated (U: HLA-dr−, BA1−, four cases) and pre-common (pre-C: HLA-dr+, BA1−, four cases or HLA-dr+, BA1+, six cases), common (C: 66 cases), thymus-derived (pre-T and T: 20 cases), immunoglobulin-positive (pre-B, CyIg+, 12 cases, and B, SIg+, 10 cases). OKT9 (purchased from Ortho), which recognises the transferrin receptor and identifies activated and/or proliferative cells, was studied in 30 adults and 36 children.

*Flow cytometry methods*

Flow cytometry study of DNA and protein content was carried out for all the patients. All determinations were performed on bone marrow aspirates. In children, bone marrow aspirates were taken under complete anaesthesia to ensure good quality of the samples. The flow cytometry technique used has been described previously (Ffrench *et al.*, 1985). Briefly, propidium iodide (PI) and fluorescein isothiocyanate (FITC) were used for simultaneous staining of DNA and proteins in ethanol-fixed cells after RNAse treatment, as described by Crissman & Steinkamp (1973). The stained cells were analysed in a Cytofluorograf H50 (Ortho Instruments, Westwood, MA). Two-variable cytograms were displayed on the screen of the flow-cytometer enabling selected zones to be defined. For each sample, 10 000 cells were analysed. The cytofluorograph was calibrated using normal peripheral blood lymphocytes in order to allow interassay comparisons.

*Statistical methods*

The percentage of cells in the cell cycle phases G0/G1, S and G2+M was calculated from the DNA histogram according to Model 1 of Baisch's method (Baisch *et al.*, 1975). As we described earlier (Ffrench *et al.*, 1987), the G0/G1 fraction was divided into two subgroups corresponding to low and high cellular protein content fractions. The low protein content fraction of G0/G1 (LPC fraction) can be considered as early G1.

In this study we used classical statistical methods. The $\chi^2$ test was used to compare the distributions of children and adults according to clinical and biological variables: sex, organomegaly, leukocytosis divided in three classes ($<10$, 10–50, $>50\times10^9\,l^{-1}$), cytology and immunology.

Multiple analysis of variance was used to compare the mean cell cycle variables between groups defined by age (children versus adults), organomegaly and biological characteristics (Pillai's test). The distributions of LPC and G2+M values did not differ significantly from the hypothesis of normality. But the distribution of S phase values presented a significant positive asymmetry (skewness $=2.2\pm0.23$). Since the distribution of the logarithm of S (log S) did not significantly diverge from the hypothesis of normality the three dependent variables LPC, log S and G2+M were studied jointly by variance analysis. Four two-way variance analyses were performed to study age paired in turn with each of the following: organomegaly, degree of leukocytosis, cytological class and immunological phenotype. The statistical software used was SPSS (Manova module).

The degree of correlation between the three variables of the cell cycle was compared in adults and children using the Kendall $\tau$ (Kendall, 1970).

By multivariate logistic regression analysis (Cox, 1970) the probability of occurrence of complete remission in adults was analysed (all but three children entered complete remission). This analysis was performed with program LR of BMDP.

Survival curves were estimated by the Kaplan and Meier method. The significance of the differences observed between the curves corresponding to two or more groups was tested with the Tarone and Ware test (Miller, 1981). The prognostic value of the cell cycle variables was studied by determining four classes for each variable. The limits of each class were defined by the three quartiles of the distributions of the cell-cycle variables (quartiles divide a distribution into four parts of an equal number of elements). For each of the three variables of the cell cycle, the significance of the differences between the four survival curves was tested by the trend test of Tarone and Ware instead of the test of heterogeneity. The survival analysis was performed by program 1L of BMDP.

### Results

*Clinical and biological characteristics*

The two sets of patients were compared according to usual criteria.

The clinical and biological characteristics of the patients are listed in Table II. Clinical features did not differ between adults and children; the distribution according to sex and organomegaly did not show any statistical difference. However, we did find, as had previous longer series, that the distribution among the cytological and immunological classes was statistically very different for adults and children.

*Cytology* The L2 group was statistically larger in adults and the L1 group was larger in children. The prognostic value of the FAB classification was found to be very different for adults and children: in children, remission and survival were statistically shorter for L2 and L3 cases than for L1 (Tarone and Ware test, $P<0.01$), while in adults only

**Table II** Comparison of clinical and biological features of the 124 children and adults in whom flow cytometry was performed (number and percentage of patients)

| Variables | Children | Adults | $\chi^2$ test significance level |
|---|---|---|---|
| Age (mean) | 63 months | 38 years | |
| Sex | | | |
| M | 45 (67%) | 38 (67%) | |
| F | 22 (33%) | 19 (33%) | |
| Organomegaly | | | |
| + | 48 (72%) | 38 (69%) | $P>0.10$ |
| − | 19 (28%) | 17 (31%) | |
| not known | | 2 | |
| WBC count | | | |
| <10 | 28 (42%) | 20 (35%) | $P=0.09$ |
| 10–50 | 26 (39%) | 16 (28%) | |
| >50 | 13 (19%) | 21 (37%) | |
| Cytology | | | |
| L1 | 55 (82%) | 28 (50%) | $P=0.0006$ |
| L2 | 9 (13%) | 24 (43%) | |
| L3 | 3 (5%) | 4 (7%) | |
| unclassified | 0 | 1 | |
| Immunology | | | |
| U+Pre-C | 0+2 (3%) | 4+8 (22%) | $P=0.0008$ |
| C | 41 (61%) | 25 (45%) | |
| Pre-B | 11 (16%) | 1 (2%) | |
| B | 4 (6%) | 6 (11%) | |
| T | 9 (14%) | 11 (20%) | |
| not done | 0 | 2 | |

L3 patients had significantly shorter remission than the other cases.

*Immunology* The T and U/pre-C phenotypes were more common in adults, while C and pre-B were more numerous in children. B ALL (S Ig+) appears to have a worse prognosis in adults and in children than the other ALLs.

Cytogenetic analysis was not done for a sufficient number of cases to permit valid comparison with flow cytometry results. For children, incontestable aneuploidy was found in 27 cases out of 67, and in five cases hyperploidy did not allow the evaluation of all the cell cycle variables. In adults, aneuploidy was found in 18 cases, of which five were too abnormal for cell cycle determination.

The distribution of leukocyte counts did not differ between the two groups of patients. The study of the prognostic value of these clinical and biological characteristics showed the relationship of WBC count with the achievement of complete remission and with the duration of survival in adults but not in children. Survival was significantly longer in adult ALL when WBC count was normal (below $10 \times 10^9 l^{-1}$) ($P<0.01$).

*Comparative study of cell kinetics in adults and children*

*Cell cycle variables.* All three variables of cell cycle were determined for 109 patients (58 children and 51 adults). The mean, median, standard deviation and range of LPC, S, log S and G2+M of the 109 patients are listed in Table III. The correlations between LPC and G2+M and log S and G2+M were not significant. However, the negative correlation between LPC and log S was highly significant ($P<0.0001$; Kendall's $\tau=-0.30$).

When considering adults and children separately we have found that:

1. The correlation between LPC and G2+M was not significant in adults or children.
2. The negative correlation between LPC and log S was highly significant in both groups of patients. It did not differ statistically between the two groups.
3. The correlation between log S and G2+M was not significant in children. But in adults Kendall's $\tau$ was significant ($t=-0.21$, $P=0.02$) (Figure 1).

*Cell cycle in relation to other clinical and biological characteristics of ALL.* This analysis was limited by the small number of patients.

Variance analysis with the dependant variables LPC. log S and G2+M gave the following results: the four interactions age × organomegaly, age × leukocyte count, age × cytology and age × immunology were not statistically significant.

**Table III** Summary of the distributions of the variables of cell cycle (109 patients: 58 children and 51 adults)

| | *LPC* (%) | *S* (%) | *Log S* (%) | *G2+M* (%) |
|---|---|---|---|---|
| Mean | 35.1 | 7.8 | 1.7 | 5.5 |
| Median | 36 | 6 | 1.8 | 6 |
| Standard deviation | 17.2 | 6.6 | 0.8 | 2.6 |
| Minimum | 2 | 0.5 | −0.7 | 0.4 |
| Maximum | 76 | 38 | 3.6 | 11 |

There was no significant differences between adults and children.

**Figure 1** Relation between the proportion of cells in S-phase and G2+M-phase. A negative correlation was found in adults ($P<0.05$) but there was no correlation between these two variables in children. In adults, the regression curve was calculated with the exclusion of one patient (L3 ALL, 16 years old) for whom the values were far out of range.

Furthermore the main effects of the factors age, organomegaly and leukocytosis were not statistically significant, whereas the main effects of the factors cytology and immunology were significant.

Pillais's multivariate test gave to the main effect of cytology a level of significance $P=0.001$. The univariate F-tests indicated that this effect concerned LPC ($P<0.001$) and log S ($P=0.005$). For immunology the level of significance of Pillais's test was 0.002. Univariate F-tests showed that here too it was LPC ($P=0.001$) and log S ($P=0.004$) that were involved.

Table IV shows the means and the standard errors of LPC, log S and G2+M according to cytology and immunology.

For cytology, the method of multiple comparisons of Scheffé, applied at a level of significance 0.05, indicated that mean LPC was significantly lower and mean log S was

**Table IV** Mean cell-cycle variables according to cytological and immunological features in the 109 patients (58 children and 51 adults) for whom all cell cycle variables were available (±standard error)

| | *Number of patients* | *LPC* (%) | *S* (%) | *Log S* (%) | *G2+M* (%) |
|---|---|---|---|---|---|
| Cytology | 109 | | | | |
| L1 | 74 | 38.4±1.9 | 7.4±0.7 | 1.7±0.1 | 5.8±0.3 |
| L2 | 29 | 32 ±3 | 6.5±0.8 | 1.6±0.1 | 4.8±0.5 |
| L3 | 5 | 7.9±2.3 | 20.1±4.8 | 2.9±0.2 | 5.6±1.1 |
| Unclassified | 1 | 24 | 6.2 | 1.6 | 4 |
| Immunology | 107 | | | | |
| U/Pre-C | 13 | 32.9±4.7 | 5.1±1.3 | 1.2±0.3 | 5.8±0.6 |
| C | 59 | 38.7±2.2 | 7.4±0.8 | 1.7±0.1 | 5.8±0.3 |
| Pre-B | 10 | 42.4±4.6 | 7.3±1.7 | 1.7±0.3 | 5.1±1.1 |
| B | 7 | 11.8±3.9 | 17.2±3.8 | 2.7±0.2 | 5.2±1.1 |
| T | 18 | 31.7±3.7 | 7.7±1.2 | 1.8±0.2 | 5 ±0.2 |

The mean variables were significantly different for L3 ALL and for the B phenotype. No differences were observed according to age.

significantly higher for ALL L3 than for ALL L1 and L2, which were not significantly different from one another.

For immunology the method of Scheffé indicated that mean LPC was significantly lower for ALL B than for ALL C or pre-B and that mean log S was higher for ALL B than for ALL U/pre-C and C. Pre-B ALL did not proliferate faster than the other groups.

There was no difference between adults and children even when we compared more specifically the L1 groups (29 adults and 55 children) or the C phenotype groups (25 adults and 41 children).

There was no relation between cell cycle variables and T9 phenotype in either adults or children. However, we did find a somewhat bimodal distribution of this phenotype in the children: eight patients showed very strong positivity for T9, while the staining was close to zero in the other cases. These eight patients did not show any cell cycle particularly but four of them were T ALL (four of the six T ALLs found in children), two were pre-B and two C ALL.

Mean S-phase in hyperdiploid cases was slightly higher than in the diploid group of children and adult ALL. These differences were below the threshold of statistical significance (8.6% versus 7.27% in children and 7.75% versus 6.5% in adults).

*Cell cycle and prognosis* While cell cycle values did not seem to differ between adults and children according to biological features at diagnosis, the prognostic significance of cell proliferation appears to be very different between adults and children. In this series cell cycle variables did not appear to have any significance for predicting the achievement of complete remission. The negative prognostic value of a high percentage of cells in LPC fraction previously observed for adults (Ffrench *et al.*, 1987) was not confirmed in this larger series, and leukocytosis appeared to be the most important criterion.

*Cell cycle and length of complete remission* Cell proliferation was not helpful for predicting length of CR in adults. We found only that B ALLs, which are the most proliferative, had the shortest CR. By contrast, for children remission was statistically longer when G2+M was higher (Tarone and Ware test, $n=60$, $t=6.27$, $P=0.012$) (Figure 2). However, no relation was found between L2 cytology (which had a worse prognosis than L1) and the proportion of cells in the G2+M phase.

*Cell cycle and length of survival* As we found earlier (Ffrench *et al.*, 1987) survival was longer for adults when G2+M was between 3.8 and 5.8% or very high, over 7.2% (quartile 3), and the prognosis was poor both with the intermediary and the lowest proliferative activity ($P<0.01$) (Figure 3). In contrast, in children survival was longer when G2+M was high (Tarone and Ware test, $n=62$, $t=7.14$, $P<0.01$) (Figure 4).

The S-phase did not appear to be of prognostic value either for length of remission or for length of survival.

**Figure 2** Cell-cycle and length of complete remission in children. Remission duration was longer for higher levels of G2+M cells ($P=0.012$). 1, G2+M<3.5%; 2, 3.5%<G2+M<5.5%; 3, 5.5%<G2+M<7%; 4, G2+M>7%.

**Figure 3** Cell-cycle and length of survival of adults. Survival was longer when G2+M was between 3.8 and 5.8% or over 7.2% ($P<0.01$). 1, G2+M<3.8%; 2, 3.8%<G2+M<5.8%; 3, 5.8%<G2+M<7.2%; 4, G2+M>7.2%.

**Figure 4** Cell-cycle and length of survival of children. The Tarone and Ware test was significant ($P<0.01$), with longer survival for higher levels of G2+M cells. 1, G2+M<3.5%; 2, 3.5%<G2+M<5.5%·; 3, 5.5%<G2+M<7%; 4, G2+M>7%.

### Discussion

The difference in prognosis between adult and childhood ALL is well known (Gee *et al.*, 1977). This difference of responsiveness to chemotherapy is not currently well understood. The distribution among cytological and immunological groups is different (Bennett *et al.*, 1981; Foà *et al.*, 1985). All the authors have found the proportion of L1 and common ALL to be higher in children than in adults, but this alone cannot explain the poor prognosis of adults. In particular, L1 and common ALL do not have a better rate of complete remission in adults than the other kinds of ALL. The present study attempted to discover whether cell cycle investigation can contribute to resolving these discrepancies.

Dosik *et al.* (1980) and Hiddeman *et al.* (1982) have shown that different S-phase values are obtained with different methods of sampling (aspiration or bone-marrow biopsy). However, in spite of the drawbacks involved in the aspiration technique, our previous findings (Ffrench *et al.*, 1986) have shown the usefulness of analysing other cell-cycle variables, particularly the LPC-fraction, which can be considered as representative of the quiescent cells and of the cells in the initial phase of the cell cycle (early G1).

In this study the cell kinetics of ALL according to clinical and biological features were compared between adults and children. We did not find age-related differences even when common ALL or L1 ALL were compared between the two groups of patients. The sole differences observed were linked to B ALL (or L3 ALL known to be more proliferative than the other kinds of ALL (Walle, 1986)). In this study, cell kinetics of undifferentiated or pre-C, of pre-B and T ALL were not significantly different from those of common ALL.

While several of the biological features of childhood ALL are well known (Sellan *et al.*, 1980; Secker-Walker *et al.*, 1982), the nature of the cell proliferation does not yet seem to be completely elucidated in spite of the extensive study it has received. Look *et al.* (1982) investigated the significance of a high percentage of cells in S phase, particularly in hyperdiploid C-ALL which has a good prognosis: does it reflect high proliferative activity or long duration of DNA replication? Suarez *et al.* (1985) showed a significantly higher incidence of aneuploidy in L2 than in L1 patients. In the L1 group both S and S+G2+M were significantly different between aneuploid and diploid ALL. Dörmer *et al.* (1984) found that in untreated childhood ALL DNA synthesis time was significantly longer than that in lymphocytes of various normal tissues. Labelling index and DNA synthesis time were slightly higher in hyperdiploid cases, but this was below the threshold of statistical significance. These differences, however, did not exist for the DNA synthesis rate. The amount of DNA to be synthesised may contribute to the differences in labelling index and DNA synthesis time between euploid and hyperdiploid ALL. In our series, due to the small number of cases, we did not observe a significant difference between diploid and hyperdiploid groups. Furthermore in five of the hyperdiploid cases the S-phase could not be calculated.

Look *et al.* (1985) demonstrated a correlation between a low percentage of S-phase cells (below 6.8%) and the lack of response to induction therapy, and took these results to indicate the need for intensifying therapy, following the example of Riehm *et al.* (1980). In our study, we found no prognostic value for the S-phase in children but all received BFM therapy and only three failed to achieve CR. Cell proliferation in adult ALL has been less studied but some questions have been raised (Ffrench *et al.*, 1987), in particular, what is the significance of the negative correlation between S and G2+M?

In ALL, the failure of remission-induction chemotherapy must be essentially related to 'drug resistant disease'. Our comparative study of cell proliferation in adult and childhood ALL focused on the proportion of cells in G2+M phase. G2+M appears to be of particular prognostic interest in children, for whom prognosis is best when G2+M is the highest. Different hypotheses can be advanced to account for the high levels of G2+M cells.

A high degree of proliferation. The negative correlation between S and G2+M observed in adults and the absence of correlation in children militates against this theory.

The existence of tetraploid clones. Look *et al.* (1982) found that some patients (22 of the 225 cases studied) presented a G2+M phase higher than expected from the S phase value, which this author interpreted as tetraploidy. However, Williams *et al.* (1982) found two karyotypes with modal numbers 90–91 in a series of 136 patients. More recently, Heerema *et al.* (1985) observed, with cytogenetic techniques, one case of tetraploidy in a series of 70 patients. When compared to cytogenetic findings, the frequency of high levels of G2+M found by Look *et al.* (1982) seems too high to be considered only as tetraploidy. However, mitosis of a small proportion of tetraploid cells may be a very rare event and tetraploidy might then be underestimated by cytogenetics.

An accumulation of cells in G2 or in the end of S phase and variations in the duration of these phases. Such an accumulation of non-cycling cells with a G2 DNA content has been described after irradiation (Rowley & Leeper, 1985) or treatment with anticancer drugs (Rao & Rao, 1976), and could be an explanation of ageing (Gelfant & Smith, 1972). This hypothesis would account for the different reactions to chemotherapy between adults and children and calls for further investigation of the cell cycle in ALL.

### References

BAISH, H., GOHDE, W. & LINDEN, W.A. (1975). Mathematical analysis of ICP – data to determine the fraction of cells in the various phases of cell-cycle. *Pulse-cytometry*, **1**, 68.

BENNETT, J.M., CATOVSKY, D., DANIEL, M.T. & 4 others (1981). The morphological classification of acute lymphoblastic leukaemia: concordance among observers and clinical correlations. *Br. J. Haematol.*, **47**, 553.

COX, D.R. (1970). *The Analysis of Binary Data*. Chapman and Hall: London.

CRISSMAN, H.A. & STEINKAMP, J.A. (1973). Rapid simultaneous measurement of DNA, protein and cell volume in single cells from large mammalian cell populations. *J. Cell. Biol.*, **59**, 766.

DOSIK, G.M., BARLOGIE, B., SMITH, T.L. & 4 others (1980). Pretreatment flow cytometry of DNA content in adult acute leukemia. *Blood*, **55**, 47.

DÖRMER, P., UCCI, G., LAU, B., HAAS, R.J. & JANKA, G.E. (1984). *In vivo* production of childhood acute lymphoblastic leukemia cells in relation to ploidy and immunological subtype. *Leukemia Res.*, **8**, 587.

DOSIK, G.M., BARLOGIE, B., GÖHDE, W., JOHNSTON, D., TEKELL, J.L. & DREWINKO, B. (1980). Flow cytometry of DNA content in human bone marrow: a critical reappraisal. *Blood*, **55**, 734.

DOW, L.W., CHANG, L.J.A., TSIATIS, A.A., MELVIN, S.L. & BOWMAN, W.P. (1982). Relationship of pretreatment lymphoblast proliferative activity and prognosis in 97 children with acute lymphoblastic leukemia. *Blood*, **59**, 1197.

FFRENCH, M., BRYON, P.A., FIÈRE, D. & 4 others (1985). Cell-cycle, protein content and nuclear size in acute myeloid leukemia. *Cytometry*, **6**, 47.

FFRENCH, M., BRYON, P.A., FIÈRE, D. & 4 others (1986). Cell-cycle prognostic value in adult acute myeloid leukemia. The choice of the best variables. *Leukemia Res.*, **10**, 51.

FFRENCH, M., MANEL, A.M., MAGAUD, J.P. & 4 others (1987). Adult acute lymphoblastic leukaemia: is cell proliferation related to other clinical and biological features? *Br. J. Haematol.*, **65**, 419.

FOÀ, R., BALDINI, L., CATTORETTI, G. & 11 others (1985). Multimarker phenotypic characterisation of adult and childhood acute lymphoblastic leukaemia: an Italian multicentre study. *Br. J. Haematol.*, **61**, 251.

GEE, T.S., HAGHBIN, M., DOWLING, M.D., CUNNINGHAM, I., MIDDLEMAN, M.P. & CLARKSON, B.D. (1976). Acute lymphoblastic leukemia in adults and children. Difference in response with similar therapeutic regimens. *Cancer*, **37**, 1256.

GELFANT, S. & SMITH, J.G. (1972). Aging: noncycling cells an explanation. Cell and tissue aging is the result of transitions from cycling to noncycling cells. *Science*, **178**, 357.

HEEREMA, N.A., PALMER, C.G. & BAEHNER, R.L. (1985). Karyotypic and clinical findings in a consecutive series of children with acute lymphocytic leukemia. *Cancer Genet. Cytogenet.*, **17**, 165.

HIDDEMAN, W., BÜCHNER, T., ANDREEFF, M., WÖRMANN, B., MELAMED, M.R. & CLARKSON, B.D. (1982). Cell kinetics in acute leukemia: a critical reevaluation based on new data. *Cancer*, **50**, 250.

HOLDRINET, R.S.G., PENNINGS, A., DRENTHE-SCHONK, A.M., VAN EGMOND, J., WESSELS, J.M.C. & HAANEN, C. (1983). Flow cytometric determination of S-phase compartment in adult acute leukaemia. *Acta Haematol.*, **70**, 369.

KENDALL, M.G. (1970). *Rank Correlation Methods*. Charles Griffin: London.

KURIYAMA, K., TOMANAGA, M., NONAKA, H. & 8 others (1985). A comparative study of immunological and cytochemical profiles between adult and childhood acute lymphoblastic leukemias (ALLs): heterogeneity in adult common ALL. *Leuk. Res.*, **10**, 1237.

LOOK, A.T., MELVIN, S.L., WILLIAMS, D.L. & 5 others (1982). Aneuploidy and percentage of S-phase cells determined by flow cytometry correlated with cell phenotype in childhood acute leukemia. *Blood*, **60**, 959.

LOOK, A.T., ROBERSON, P.K., WILLIAMS, D.L. & 9 others (1985). Prognostic importance of blast cell DNA content in childhood acute lymphoblastic leukemia. *Blood*, **65**, 1079.

MILLER, D.R., LEIKIN, S., ALBO, V., SATHER, H. & HAMMOND, D. (1981). Prognostic importance of morphology (FAB classification) in childhood acute lymphoblastic leukaemia (ALL). *Br. J. Haematol.*, **48**, 199.

MILLER, R.G. (1981). *Survival Analysis*. Wiley: New York.

MURPHY, S.B. AUR, R.J.A., SIMONE, J.V., GEORGE, S. & MAUER, A.M. (1977). Pretreatment cytokinetic studies in 94 children with acute leukemia. Relationship to other variables at diagnosis and to outcome of standard treatment. *Blood*, **49**, 683.

NADLER, L.M., KORSMEYER, S.J., ANDERSON, K.C. & 9 others (1984). B cell origin of non-T cell acute lymphoblastic leukemia. A model for discrete stages of neoplastic and normal pre-B cell differentiation. *J. Clin. Invest.*, **74**, 332.

PREISLER, H.D., AZARNIA, N., RAZA, A. & 10 others (1982). Relationship between the percent of marrow cells in S-phase and the outcome of remission-induction therapy for acute non lymphoblastic leukaemia. *Br. J. Haematol.*, **56**, 399.

RAO, A.P. & RAO, P.N. (1976). The cause of G2-arrest in Chinese hamster ovary cells treated with anticancer drugs. *J. Natl Cancer Inst.*, **57**, 1139.

RIEHM, H., GADNER, H., HENZE, G., LANGERMANN, H.J. & ODENWALD, E. (1980). The Berlin childhood acute lymphoblastic leukemia therapy study, 1970–1976. *Am. J. Pediatr. Hematol. Oncol.*, **2**, 299.

ROWLEY, R. & LEPPER, D.B. (1985). Cell cycle age dependence for radiation-induced G2 arrest: evidence of time-dependent repair. *Radiat. Res.*, **103**, 326.

SALLAN, S.E., RITZ, J., PESANDO, J. & 5 others (1980). Cell surface antigens: prognostic implications in childhood acute lymphoblastic leukemia. *Blood*, **55**, 395.

SCARFFE, J.H., HANN, I.M., EVANS, D.I.K. & 4 others (1980). Relationship between the pretreatment proliferative activity of marrow blast cells and prognosis of acute lymphoblastic leukemia of childhood. *Br. J. Cancer*, **41**, 764.

SECKER-WALKER, L.M., SWANSBURY, G.J., HARDISTY, R.M. & 4 others (1982). Cytogenetics of acute lymphoblastic leukaemia in children as a factor in the prediction of long-term survival. *Br. J. Haematol.*, **52**, 389.

SUAREZ, C., MILLER, D.R., STEINHERZ, P., MELAMED, M.M. & ANDREEF, M. (1985). DNA and RNA determination in 111 cases of childhood acute lymphoblastic leukaemia (ALL) by flow cytometry: correlation of FAB classification with DNA stemline and proliferation. *Br. J. Haematol.*, **60**, 677.

WALLE, A.J. (1986). Identification of L3 leukemia and Burkitt's lymphoma cells by flow cytometric quantitation of nuclear and cellular RNA and DNA content. *Leuk. Res.*, **10**, 303.

WILLIAMS, D.L., TSIATIS. A., BRODEUR, G.M. & 6 others (1982). Prognostic importance of chromosome number in 136 untreated children with acute lymphoblastic leukemia. *Blood*, **60**, 864.